# An insight into the salivary gland content of the human body louse, *Pediculus humanus*

**DOI:** 10.1038/s41598-025-01412-5

**Published:** 2025-05-26

**Authors:** David M. Bland, Stephen Lu, Sazzad Mahmood, José M. C. Ribeiro

**Affiliations:** 1 Laboratory of Bacteriology, National Institute of Allergy and Infectious Diseases, Hamilton, MT USA; 2 Vector Biology Section, Laboratory of Malaria and Vector Research, National Institute of Allergy and Infectious Diseases, Rockville, MD USA; 3 Tick-Pathogen Transmission Unit, Laboratory of Bacteriology, National Institute of Allergy and Infectious Diseases, Hamilton, MT USA

**Keywords:** RNA sequencing, Sialome, Transcriptome, Hematophagous arthropods, Vector, Salivary gland, Louse, Entomology, Proteomics, Transcriptomics

## Abstract

Human body lice, *Pediculus humanus humanus*, are blood-feeding parasites that live in clothing and feed several times per day. Saliva injected during louse feeding induces pruritis and local inflammation in the skin. If untreated, chronic Pediculosis can cause systemic negative health effects. Despite the medical importance of body lice and their longstanding association with humans, characterization of their saliva has been limited. To address this, we extracted RNA and protein from two of the body louse’s morphologically distinct sets of salivary glands (Bean-shaped and U-shaped) and generated transcript and protein profiles for each. Additionally, we performed fluorescent staining and confocal microscopy on each gland type to enhance descriptions of their structure and gross cellular architecture. Analysis of body louse salivary gene products and proteins revealed that the overwhelming majority were not closely related to biomolecules of known function, highlighting the organism’s unique and understudied saliva composition. Despite the contrasting morphology of the two gland types, there was a high degree of overlap in the salivary products produced. This finding suggests strong Darwinian selection pressure to maintain both salivary gland types, given that it would be simpler to have a single morphologically identical set of glands. Here we present the first next-generation sequencing and proteomic characterization of the human body louse sialome, discuss the potential physiological importance of louse salivary proteins, and consider possible explanations for why lice have a complex salivary gland system despite inordinate redundancy in the protein repertoire of the Bean- and U-shaped salivary glands.

## Introduction

Human body lice (*Pediculus humanus humanus*) are blood-feeding ectoparasites that reside within the seams of clothing. Body lice feed every few hours, preferentially targeting the arm pits, shoulders, back, waistline, and thighs^1,2^. Estimates of body louse prevalence amongst homeless populations in large cities range from 19 to 68%^3,4^. Few studies enumerate the number of lice present on infested individuals, likely due to the challenging nature of collecting such data. However, in one study amongst homeless individuals in Marseilles, France, the average was 58 per person^5^. In severe cases of untreated pediculosis, individuals may harbor hundreds of lice^6,7^. Given the body louse’s fecundity and frequent feeding behavior, parasitized individuals are repeatedly exposed to a considerable amount of louse saliva.

For most people and within several hours of louse bite, small red papules form which are irritating for several days^8^. The severity of irritation is variable but generally induces significant itching^8,9^. If symptoms go untreated, abrasions develop from the ensuant scratching and provide an entry point for louse feces that can potentially contain bacterial louse-borne pathogens such as *Bartonella quintana* (trench fever), *Borrelia recurrentis* (louse-borne relapsing fever), *Rickettsia prowazekii* (epidemic tyhpus), and possibly *Yersinia pestis* (plague)^10,11^. Even in the absence of louse-borne pathogens, chronic pediculosis can result in headaches, anemia, low-grade fever, fatigue, and formation of diffuse rashes^6–8,12^.

Unlike most blood-feeding arthropods, which generally have one or two identical pairs of salivary glands, human lice have at least two, and possibly three, morphologically unique sets of salivary glands. Body lice have two pairs of recognized salivary glands: a pair of Bean-shaped (reniform) glands and an additional set of U-shaped, tubular glands^13,14^. Both sets exist in the insect’s thorax and likely account for the bulk of saliva production based on their size and anatomical interfacing with the salivary channel in the louse mouthparts. Lice have an additional 3^rd^ set of minuscule, putative accessory glands located within the head and termed the Pawlowsky glands (PGs). The potential secretions produced by the PGs are hypothesized to lubricate the mouthparts during their extension and retraction and it’s unknown whether they produce proteins with utility at the feeding site in the skin^15^.

Scientific literature exploring the pharmacological activity of *P. humanus* salivary gland products has been limited, with only a few studies examining extracts from both Bean- and U-shaped glands^13,16^. Although such studies demonstrated vasodilatory and anticoagulant activity for *P. humanus* salivary gland homogenates, it was not possible to identify gland specificity nor the molecules responsible.

In order to catalog and characterize the body louse Bean- and U-shaped gland sialomes we performed RNA-sequencing followed by mass spectrometry analysis to confirm the transcripts and proteins produced by both glands. We show that the Bean- and U-shaped glands have a high degree of redundancy in the salivary proteins they produce and most of these did not have known function. Salivary protein families typically found in other blood-feeding arthropods that was also identified in body louse saliva included peptidases, peptidase inhibitors, and antigen-5 like proteins. The data presented here will serve as a guide for targeting human body louse salivary proteins for pharmacological characterization and development of recombinant antigens that may serve as immunological markers of *P. humanus* exposure.

## Materials and methods

### Body louse rearing and salivary gland collection

Frisco-BL strain (San Francisco) human body lice^17^ were reared as described previously^11^. To collect salivary glands, mixed-sex adult lice were collected from their housing chambers and immobilized by storing on ice for 10 min. Individual lice were transferred to a drop of ice-cold RNase-free phosphate buffered saline (PBS; Invitrogen) on a microscope slide using fine forceps. Next, both the head and most of the abdomen were removed using micro-scissors. The mass of tissue remaining within the thorax was removed with forceps and the Bean- and U-shaped glands were isolated from non-target tissues and transferred to new, individual drops of PBS.

For bulk RNA extraction, glands were transferred to individual 1.5 ml Eppendorf tubes containing 200 µl of RNAlater (Invitrogen, Waltham, MA) and frozen at −80 °C immediately following collection. Forceps were rinsed in sterile PBS and cleaned with a Kimwipe prior to transfer of the salivary glands. Collections were performed on 5 separate occasions until a cumulative 200 individual glands of each type had been collected and frozen. Two independent sets of salivary gland samples were collected for RNA extraction and sequencing. For mass spectrometry, glands were collected as described above but were stored in PBS prior to freezing.

### Salivary gland staining and microscopy

For light microscopy, salivary glands were isolated as described above, placed in a drop of PBS, and overlaid with a glass coverslip. Glands were imaged using a Nikon SMZ1500 dissection microscope with a DP72 Olympus camera.

For fluorescent staining and imaging, 20 salivary glands of each type were collected and transferred to a 1.5 ml Eppendorf tube containing 200 µl of PBS with 0.25% bovine serum albumin prior to staining. Glands were initially stained with 0.1% Malachite Green oxalate (Fisher Scientific, Hampton, NH) for 10 min to aid in their visualization and retention. Unbound, excess stain was removed by three washes with PBS. Glands were then fixed with 4% paraformaldehyde for 1 h followed by permeabilization with 0.1% Triton-X 100 for 30 min. Filamentous actin and DNA were stained simultaneously by incubating the samples with 2 units of Alexa Fluor™ 488 Phalloidin (Invitrogen) dissolved in 400 µl of 4 µM Hoechst 33342 (Thermo Scientific, Waltham, MA) for 30 min. After each step, samples were washed twice with PBS. All steps were performed at room temperature. To avoid inadvertently removing and discarding salivary glands, samples were gently spun in a minicentrifuge for 2 min to pool glands near the bottom of the tube and only ~ 80–90% of the total volume (800–900 µl) was removed and replaced in between each step.

After staining, samples were transferred to a Poly-D-Lysine coated 35 mm petri dish (MatTek, P35GC-1.5-14-C) for imaging. Glands were imaged using a Zeiss laser scanning confocal microscope (LSM 880) equipped with 405 nm and 488 nm laser lines and the ZEN v. 2.3 software (Carl Zeiss Microscopy). Images were acquired with a Plan-Apochromat 20X/NA0.8 lens and stacks were collected with a z-spacing of 1 µm.

### Protein extraction and silver staining

For protein visualization, 100 pairs of Bean- and U-shaped salivary glands were pooled and homogenized in PBS (pH 7.2) using a sterile pestle (Sigma, USA). The homogenate was centrifuged at 10,000×*g* for 10 min at 4 °C, and the supernatant was collected. Total protein concentration was determined using the BCA Protein Assay Kit (Pierce, USA) according to the manufacturer’s instructions. Approximately 5 µg of total protein from Bean-shaped glands and 3 µg from U-shaped glands were loaded onto a 10% SDS-PAGE gel. The gel was then stained using the Silver Stain for Mass Spectrometry Kit (Pierce, USA) following the manufacturer’s protocol.

### RNA extraction, Illumina sequencing and data analysis

Total RNA from *P. humanus* Bean-shaped or U-shaped salivary glands were isolated using the AllPrep DNA/RNA/Protein Mini Kit (QIAGEN) according to the manufacturer instructions and RNA integrity and quantity was assessed using the RNA Nano 6000 Assay Kit of the Bioanalyzer 2100 system (Agilent Technologies, CA, US). Directional mRNA libraries were prepared with poly A enrichment and sequenced using an Illumina Novaseq 6000 DNA sequencer (Illumina). The raw Illumina reads were trimmed of adapters and any low-quality sequences (Q < 20) using TrimGalore (https://github.com/FelixKrueger/TrimGalore), merged into a single file and assembled using ABySS (V2.3.1)^18^ with k values from 25 to 95 (with increments of 10) in single-stranded mode and with Trinity (V2.9.0)^19^ in single-stranded F mode. The assemblies from ABySS and Trinity were combined and filtered using CD-HIT^20^. Coding DNA sequences (CDS) with an open reading frame (ORF) of at least 150 nucleotides were extracted based on BLASTp results to several databases, including the transcriptome shotgun assembly (TSA), Refseq-invertebrate, and a subset of the non-redundant protein databases. CDS were extracted if sequences had at least 70% coverage with a matching protein. Additionally, all ORFs starting with a methionine and 40 amino acids in length were submitted to the signalP tool (V3.0). Sequences with a putative signal peptide were mapped to the ORFs and the most 5’ methionine codon was selected as the transcript start site^21^. The extracted CDS from our de novo assembly were merged with those annotated in the Orlando (Culpepper) strain body louse genome^22^ (GenBank GCA_000006295.1) and redundant sequences were filtered using the CD-HIT tool (95% of identity). For annotation we used an in-house program that scans a vocabulary of ~ 400 words and their order of appearance in the protein matches from BLAST results against several databases (TSA, subset of the NR, Refseq-protozoa, Refseq-invertebrate, Refseq-vertebrate, uniprot, MEROPS, PFAM, Smart and CDD), including their e-values and coverage. Relative quantification of CDS was performed by mapping the trimmed library reads to the extracted CDS using the RSEM tool^23^. The annotated CDSs were exported to a hyperlinked Excel spreadsheet available for download (Supplementary file 1). Protein structure prediction was performed using AlphaFold2^24^. Heatmap plots and bar graphs were generated using *pheatmap* and *ggplot2* packages for R^25^.

### Mass spectrometry analysis

For mass spectrometry analysis, 200 Bean- and U-shaped salivary glands from adult *P. humanus* were pooled and homogenized in PBS pH 7.2 using a sterile pestle (Sigma, USA). Samples were centrifuged (10,000×*g*, for 10 min at 4 °C), supernatant was collected, and total protein concentration was determined using a BCA protein assay. An aliquot of the salivary gland homogenate containing 3 μg of total protein was diluted in 50 mM HEPES pH 8.0 to a final volume of 30 μl. Protein was reduced using 5 mM dithiothreitol for 40 min at 37 °C. Samples were cooled to room temperature and alkylated with 15 mM iodoacetamide for 20 min. Then, 200 ng of trypsin was added, and samples were incubated for 15 h at 37 °C. The pH was adjusted to approximately 2.5 with 10% trifluoroacetic acid (TFA) and samples were desalted and concentrated with Agilent OMIX10 tips (Agilent, USA). Peptides were eluted with 20 μl of 0.1% TFA/50% acetonitrile (ACN) and dried under vacuum. The peptides were dissolved in 12 μl of 0.1% formic acid (FA)/3% ACN and centrifuged at 18,000×*g* for 5 min. The LC–MS experiment was performed using an Orbitrap Fusion Lumos mass spectrometer (Thermo Fisher Scientific, USA) coupled to the EASY nLC 1200 nano-liquid chromatography system (Thermo Fisher Scientific, USA). Peptides were first bound to a PepMap C18 column (3 μm particle, 100 Å pore, 75 μm inner diameter, 2 cm length) then separated using an EASY-Spray analytical column (PepMap C18, 2 μm particle, 100 Å pore, 75 μm inner diameter, 25 cm length) using a linear gradient of 0 to 40% ACN containing 0.1% FA for 100 min, followed by 40–80% for 5 min, 80% hold for 5 min, 80–0% for 5 min, and 0% hold for 5 min, with a flow rate of 200 nl/min. Data were acquired using a standard data-dependent acquisition strategy, in which the survey MS1 scan was done at least every 3 s with Orbitrap mass analyzer at 120,000 resolution. The MS2 scans were done with a linear ion trap mass analyser set for multiply charged precursor ions isolated with a 1.6 m/z window using a quadrupole and fragmented by CID at 35% collision energy. The EASY-IC internal calibration was utilized for Orbitrap scans, and the dynamic exclusion period was set at 60 s. Tandem mass spectra were analysed using the PatternLab for proteomics 4.0 platform^26^. A target-decoy database was generated using the CDS obtained from RNAseq analysis, searched using the Comet tool^27^, and implemented in PatternLab. The search space included all semi-tryptic peptide candidates and carbamidomethylation of cysteine was used as a static modification. Data were searched with a 20 ppm precursor ion tolerance and a 0.4 Da fragment ion tolerance. The validity of the peptide spectrum matches (PSMs) generated by Comet was assessed using the Search Engine Processor (SEPro) module from PatternLab. A cutoff score was established to accept a protein false discovery rate (FDR) of 1% based on the number of decoys. Results were post-processed to only accept PSMs with < 10 ppm precursor mass error, and only proteins with at least one unique peptide were considered. The normalized spectral abundance factor (NSAF) was used to measure relative abundance of proteins.

## Results and discussion

### Human body louse salivary glands

**Fig. 1:**
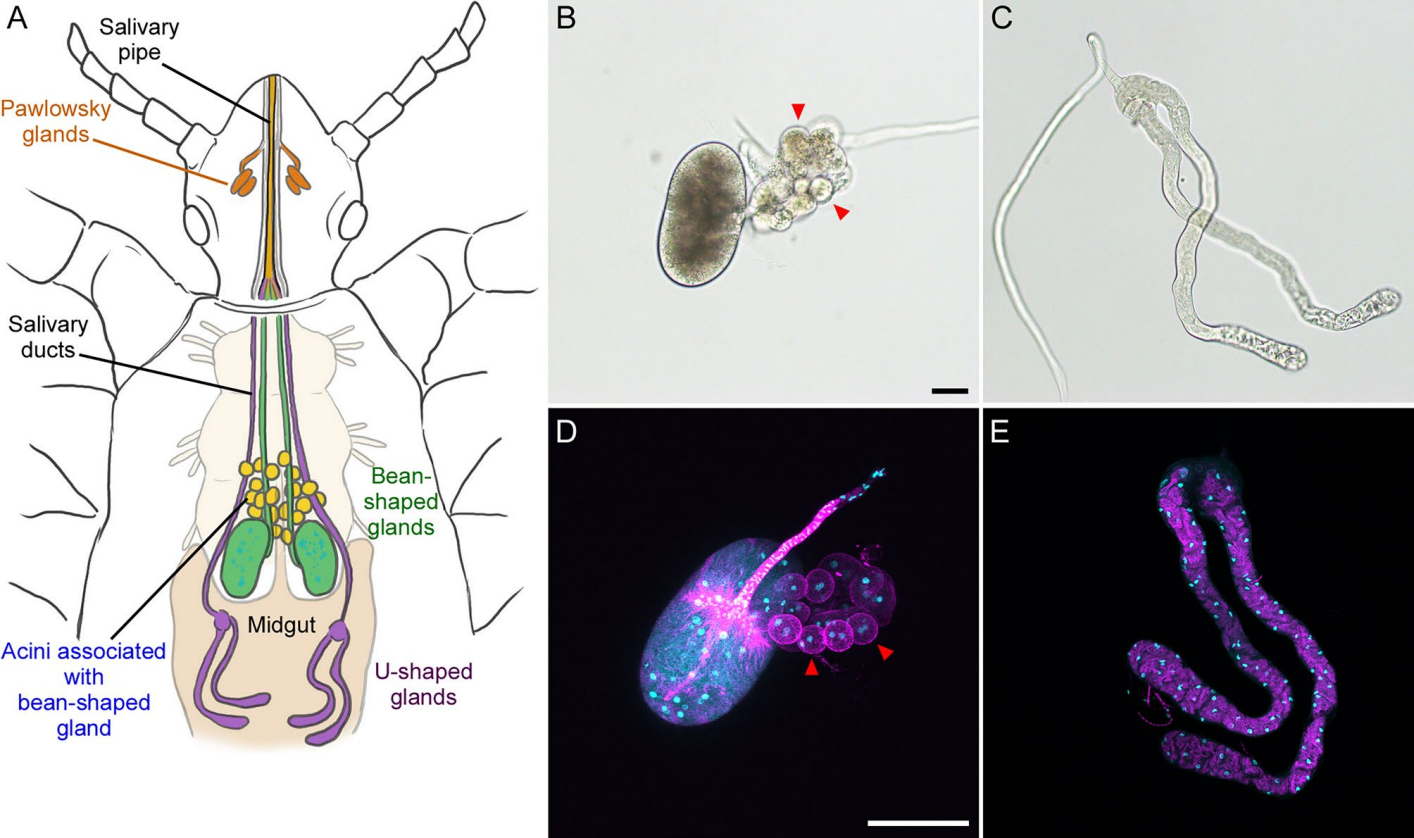
Human body louse salivary glands. (**A**) Diagram of the human body louse salivary glands and their anatomical locations. Salivary proteins produced in the Bean- and U-shaped glands in the thorax are trafficked through the salivary ducts which converge into a single salivary pipe that traverses the length of the body louse mouthparts prior to being ejected during blood-feeding. The Pawlowsky glands (PG) are putative salivary glands hypothesized to generate secretions that lubricate the louse mouthparts. The PG ducts do not interface with the salivary pipe. Light microscopy and fluorescent confocal microscopy images of the (**B**, **D**) Bean- or (**C**, **E**) U-shaped salivary glands. Fluorescent images show DNA (teal) and actin (magenta) in fixed glands stained with Hoechst and Phalloidin. Red arrows denote the acini associated with the Bean-shaped gland. Scale bars are equivalent to (**B**) 50 and (**D**) 100 µm.

The Bean-shaped and U-shaped salivary glands of body lice are located within the insect’s thorax (Fig. 1A) and each gland has a dedicated salivary duct^13,14^. All four ducts converge and feed into the single channeled salivary pipe residing within the louse’s extendable piercing-sucking mouth parts^15^. The Pawlowsky glands (PGs), located in the head, physically reside above and about half-way along the length of the salivary pipe (Fig. 1A), and, rather than interfacing with it, the ducts of the PGs feed into grooves at the bottom of the chamber housing the mouthparts^15^. Given the differential pathing of potential PG secretions, it’s uncertain of what role they may play at the feeding site in the skin. Unfortunately, due to the small size of the PGs and our inability to readily excise them from the louse head, we were unable to isolate them for transcriptomic and proteomic analysis.

The Bean-shaped glands have two major features: The salivary duct and the Bean-shaped (reniform) structure. The main reniform gland is multinucleated with dense actin staining at the base of the salivary duct (Fig. 1B and D). Based on its size, morphology, and direct connection to the salivary duct we would predict that this structure produces the majority of the body louse’s saliva. This view is supported by our recovery of about 3 times as much RNA and protein from equivalent numbers of the Bean-shaped glands compared to the U-shaped glands. There is an associated grape-like cluster of cells (acini) that resides near the base of the main salivary duct that we hypothesize is a unique organ with a function distinct from saliva production. Anatomical illustrations and descriptions of the salivary system of lice typically do not describe nor consider the acini as part of the salivary glands^14^, though one report suggests otherwise^13^. The clusters contain approximately 15 individual acinus, with some heterogeneity in size, each of which typically house 2 nuclei (Fig. 1D). Some of the acinus appear to be attached near the intersection of the salivary duct with the gland, however, the majority of the acinus attach to one another and not the Bean-shaped gland or its main duct (Fig. 1B and D). The louse acini do not have secondary ducts leading from individual acinus to any other structure; unlike the acini of tick salivary glands, where each acinus has its own individual duct arranged in an elaborate, branching network^13,28^. Because there is no obvious connection between acini and the gland to facilitate transport of salivary secretions (Fig. 1D), we think it unlikely that the acini produce saliva. Based on our observations and the ambiguity of this tissue’s designation in the literature, we did not consider the acini as part of the Bean-shaped gland for this study and removed and discarded the tissue during sample collection.

The U-shaped tubular glands are seemingly not as complex as their Bean-shaped counterpart. Though longer than the Bean-shaped glands, they are smaller in volume (Fig. 1C and E). Cell nuclei and actin are evenly dispersed along the gland’s entire length, even within the slightly wider lobed ends near the open portion of the U shape. The salivary duct intersects with the gland at the opposite, closed end of the U.

### Overview of *P. humanus* sialotranscriptome and sialoprotome

The Illumina-based RNA-sequencing of *P. humanus* Bean- and U-shaped salivary glands resulted in 78,610,978 and 78,229,280 high quality (Q > 20) reads, respectively. Following our de novo assembly and CDS extraction pipeline, we identified a cumulative 15,728 unique putative CDS, which were then integrated with the coding sequences already annotated in the existing *P. humanus* Orlando strain genome (GenBank GCA_000006295.1)^22^. We further consolidated transcripts with at least 95% identity, resulting in a total 20,822 sequences.

When aligning the trimmed library reads to these filtered transcripts, we observed an alignment rate of 15.06 ± 0.52% for the Bean-shaped libraries and 41.97 ± 4.75% for the U-shaped ones. Despite the relatively low mapping rates for the Bean-shaped samples, our evaluation using the Benchmarking of Universal Single Copy Orthologs (BUSCO) against the Insecta database indicated a high completeness of our dataset, with 80.7% complete (75.9% single and 4.8% duplicate), 4.0% fragmented and 15.3% missing. This suggests that the presence of a high number of unmapped reads is not directly linked to sample quality but is possibly attributable to the abundance of reads originating from the 5’ or 3’ untranslated regions (UTR) removed during our CDS prediction pipeline. We cannot exclude the possibility that a portion of the unmapped reads potentially originate from CDS that are not annotated in the current *P. humanus* genome or were not extracted due to the lack of a putative signal peptide, similarity to previous deposited sequences, or had open reading frames with less than 200 nucleotides, which includes non-coding RNAs. The U-shaped salivary glands had similar BUSCO results, with 87.1% complete, 4.2% fragmented, and 8.7% missing, consistent with BUSCO results from the sialomes of other blood feeding arthropods^29–31^.

**Fig. 2:**
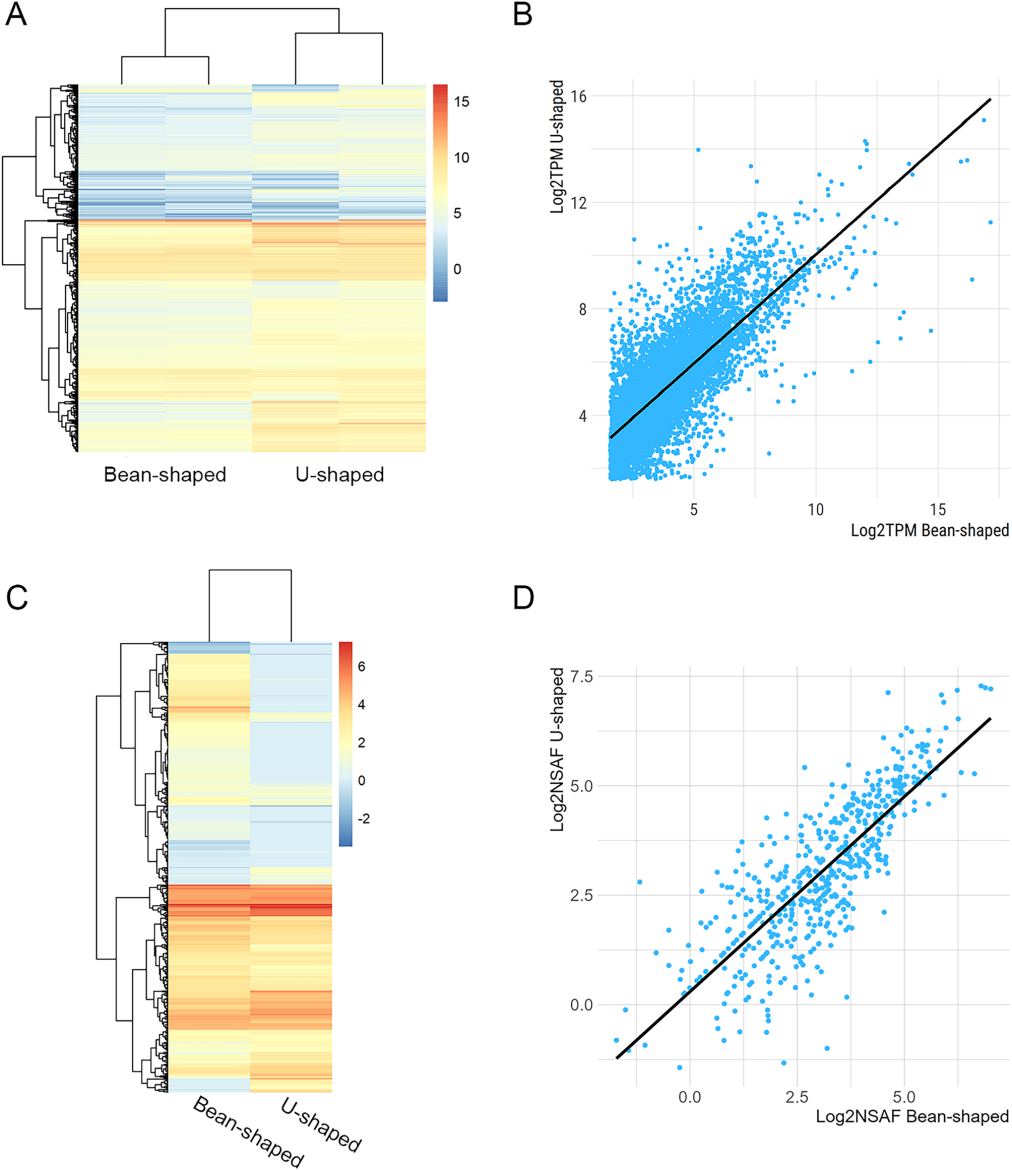
Overview of *P. humanus* Bean- and U-shape salivary glands transcriptomic and proteomic analysis. (**A**) Heatmap plot of the Log_2_TPM values from the 10,944 transcripts with an average TPM value of at least 3 for two biological replicates of each gland. (**B**) Scatterplot of the 7,505 transcripts shared between both salivary glands. A general linear model was fitted to the data (line) and the Pearson correlation coefficient (R^2^) estimated at 0.78. (**C**) Heatmap plot of the Log_2_NSAF of the 911 (Bean-shaped) and 604 (U-shaped) proteins identified in the LC–MS/MS analysis. (**D**) Scatterplot of the Log_2_NSAF from the 568 proteins common to both salivary glands. A general linear model was fitted to the data (line) and the Pearson correlation coefficient (R^2^) estimated at 0.8.

For downstream analysis, we focused on CDS with an average TPM (transcripts per million) value of at least 3 in either Bean-shaped (8,034 CDS) or U-shaped (10,315 CDS) glands, resulting in a combined set of 10,944 putative CDS (Fig. 2A). Unexpectedly, we observed a strong Pearson correlation coefficient (R^2^ = 0.7775) between the Log_2_TPM values of 7,405 transcripts that were shared between both salivary glands (Fig. 2B). This robust correlation also indicates that the salivary gland transcripts were expressed at similar levels in both gland types.

In parallel to the RNA-sequencing we also compared *P. humanus* salivary gland homogenate composition using proteomics. After filtering *matches* that presented at least one unique peptide, we identified 916 proteins in the Bean-shaped and 596 proteins in the U-shaped SG (Fig. 2C and Supplementary file 2). Of these, 538 were shared between both tissues. Similar to the RNA-sequencing data, we observed a high Pearson correlation (R^2^ = 0.8083, Fig. 2D) between the Log_2_NSAF of the shared proteins. Silver staining of salivary gland extracts also showed similar protein profiles between the two gland types, consistent with both RNA sequencing and mass spectrometry results (supplementary Fig. 1). This finding seems to differ from a previous report showing some heterogeneity between the abundant proteins extracted from the two gland types^13^. The Orlando reference strain of body lice, adapted to feed on rabbits^32^, was used in the previous study, whereas we used the Frisco strain lice which has been adapted to feed through an artificial membrane on human blood^33^. In other blood-feeding arthropods, feeding on the blood of different mammalian hosts can alter the salivary protein repertoire^34^ and this may partially explain differences in salivary protein profiles between louse strains.

**Fig. 3:**
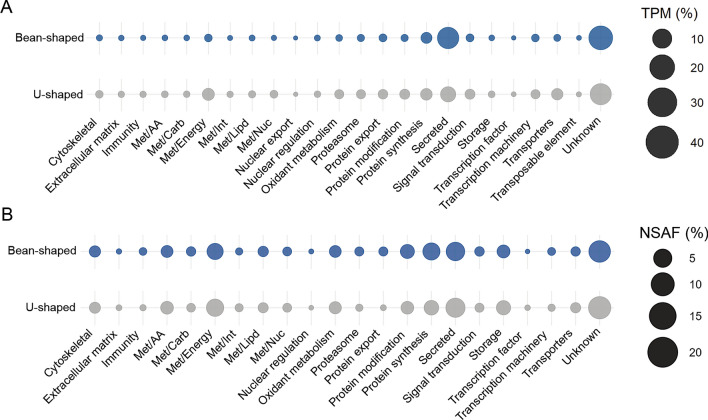
Bubble plot representing the functional classification of the transcripts and proteins identified in the Bean- and U-shaped salivary glands of *P. humanus*. The size of the spheres represents the average TPM of the CDS of each class (**A**) or the NSAF of the proteins identified by LC–MS/MS (**B**) as percentages.

To obtain a more comprehensive understanding of *P. humanus* salivary composition, we performed functional classification of the combined 10,944 transcripts (Supplementary file 1), resulting in the distribution of the CDS into 24 functional groups (Fig. 3A). For both Bean- and U-shaped salivary glands, the most prevalent functional class was the “unknown”, making up approximately 45% and 39% of the total TPM in the Bean- and U-shaped samples, respectively. Transcripts in this category have limited or no similarity to previously deposited sequences and are potentially novel. They may also share significant similarity with deposited sequences of unknown function. Overall, the high abundance of this category highlights the limited existing knowledge regarding composition and function of body louse salivary proteins.

The second most abundant class in each salivary gland was the “secreted” functional group, constituting approximately 35% of the transcripts in the Bean-shaped glands and 16% in U-shaped glands (Fig. 3A). This category includes sequences with a putative signal peptide which are typical of proteins secreted into the vector’s saliva and subsequently the host. Secreted proteins frequently exhibit potent pharmacological activity at the feeding site and may interfere with immune and hemostatic responses^35–37^. In both Bean- and U-shaped salivary glands, most of the putative secreted proteins were further subclassified as “unknown” (Table 1), however we identified several putative transcripts encoding for proteins similar to cysteine and serine peptidases, peptidases inhibitors, esterases, lipases, lipoproteins, antigen-5, and phosphatases, which are commonly reported in the sialome of other blood-feeding arthropods, such as ticks^30,38^, mosquitoes^39,40^, fleas^31,41^, and triatomines^42,43^. The third most abundant functional class in Bean-shaped salivary glands was the “protein synthesis” group, accounting for 8% of the total TPM, while in U-shaped salivary glands, this 3^rd^ position was occupied by “energetic metabolism” (Met/Energy) at 9% (Fig. 3A).

**Table 1:** Protein families within the “secreted” functional class identified in the Bean- and U-shaped salivary glands from *P. humanus*.

Protein family	Bean-shaped		U-shaped	
	No. of CDS	Average TPM (%)	No. of CDS	Average TPM (%)
27 kDa hemolymph protein	1	0.0552	1	0.0800
Angiotensin-converting enzyme	2	0.0025	3	0.0330
Amino peptidase	0.5	0.0001	0.5	0.0029
Antigen-5	2	0.0012	2	0.0141
Cysteine peptidase	15	0.1170	15	2.0617
Endothelin-converting enzyme	2	0.0040	2	0.1128
Esterase	7	0.0460	1	0.0075
Hormone binding	5	0.0105	6.5	0.1322
Lipase	2	0.0027	8	0.4943
Lipoprotein	4	0.0057	4	0.0299
Metallopeptidase	17	0.0849	16.5	0.3733
Mucin	9.5	9.1678	9.5	6.6349
Peptidase inhibitors	12	0.2060	11.5	7.6945
Phosphatase	2	0.0489	2	0.0590
Serine peptidase	31.5	0.2633	33.5	9.1485
Unknown	734.5	89.9791	808.5	73.0895

When comparing the functional classes of proteins identified by LC–MS/MS, we observed a similar overall profile (Fig. 3B). In the Bean-shaped gland, the three most abundant categories were “unknown” (21.8%), “secreted” (14.9%), and “protein synthesis” (12.1%). While in the U-shaped salivary gland, “unknown” (23.9%), “secreted” (16.4%), and “Met/Energy” (12.4%) were predominant. This observation is consistent with the substantial overlap between transcripts and proteins classes produced within both salivary gland types. Our analysis of the functional classification of transcripts and proteins unique to Bean- and U-shaped glands did reveal some differences between the two tissues. We observed that unique transporter transcripts were more prevalent in U-shaped salivary glands, while unique protein synthesis, modification, export, and proteasome proteins were more common in the Bean-shaped salivary glands (Supplementary Fig. 2).

In summary, the data presented underscores the shared core protein composition between Bean- and U-shaped glands of the Frisco strain body lice (Figs. 2 and 3). Additionally, our findings highlight the prevalence of putative secreted proteins likely injected into the host during feeding. Given their potential importance in blood acquisition, the following sections will primarily discuss families of secreted proteins identified in our analysis with predicted function.

### Peptidases

Peptidases from various families are frequently reported in the salivary glands of blood-feeding arthropods^44^. Typically, they are found with low TPM values and have not been extensively studied in the context of blood-feeding. We identified several CDS encoding putative cysteine, serine, and metallopeptidases in both Bean- and U-shaped salivary glands (Table 1). Similar to other sialomes, and despite the considerable number of CDS identified (15 cysteine, 31–33 serine and 17 metallopeptidases), most CDS had relatively low TPM values, with the exception of some serine peptidases exhibiting TPM values ranging from 1000 to 3000 (Supplementary file 1). However, our LC–MS/MS analysis identified unique peptides from several cathepsin L-like and chymotrypsin-like peptidases in both salivary gland homogenates, indicating moderate abundance.

Only a handful of salivary peptidases from blood-feeding arthropods have been shown to be involved in blood acquisition. Triapsin, a trypsin-like peptidase isolated from *Triatoma infestans* saliva, has been implicated in cleaving members of the Proteinase Activated G protein-coupled Receptors (PAR) superfamily based on substrate specificity^45^. Partially purified native triapsin preferentially cleaves PAR-2 and can induce relaxation of mouse arteries^46^. It’s believed triapsin contributes to kissing bug blood acquisition by promoting vasodilation through PAR-2 activation. Another serine peptidase named IXOSP has been purified from the saliva of *Ixodes scapularis* ticks. IXOSP can activate protein C, a potent anticoagulant, and potentially inhibit host hemostasis^47^.

Peptidases typically exhibit highly specific activity and can exert their function even in low concentrations. Despite not being overly abundant in the saliva of blood-feeding vectors, peptidases likely play essential physiological roles in arthropod blood feeding.

### Putative peptidase inhibitors

Cysteine and serine peptidase inhibitors typically act to disrupt host responses like blood clotting, platelet aggregation, and complement activation^44^. We identified multiple body louse transcripts encoding putative inhibitors of cysteine and serine peptidases (Table 1). Of these 13 putative peptide inhibitors, we identified a cystatin, a pacifastin, an alpha-2 macroglobulin, and 10 serpin family members. While most of these CDS were found in both salivary glands, 11 were more highly expressed in the U-shaped salivary glands. Four of these, seqSigP-82109, XP_002432611.1, Phseq_645, and seqSigP-2616, were also found in both glands by mass spectrometry analysis (Supplementary file 2).

The sequence seqSigP-82109 encodes a putative cystatin, a member of the tight-binding cysteine peptidase inhibitor superfamily^48^. Based on its primary sequence features, including molecular weight, presence of signal peptide and disulfide bridges, seqSigP-82109 can be further classified as a type-2 cystatin. This CDS was identified in both salivary gland types, although with higher expression levels in the U-shaped gland. Type-2 salivary cystatins in ticks modulate host immune responses by interfering with cytokine production and T-cell proliferation^49–51^. Notably, knock-down of salivary cystatin in ticks has detrimental effects on tick feeding success, including increased mortality, early detachment from the host, and a reduction in the final engorged body weight^52^. Although direct biochemical evidence is needed, it’s likely that seqSigP-82109 inhibit the activity of several cysteine peptidases to elicit their function, potentially acting similarly to tick cystatins.

Two other peptidase inhibitors, identified by LC–MS/MS, encode putative serine peptidase inhibitors belonging to the serpin and pacifastin families. The role of several salivary serpins in modulating host blood clotting^53,54^ and immunity^55–58^ has been extensively characterized in ticks and mosquitos. Antihemostatic activity by *P. humanus* salivary gland extracts has been demonstrated previously^16^, although it’s unclear whether Bean-, U-shaped, or both glands were used. The study also identified a potent 7 kDa thrombin inhibitor, however, we did not find a thrombin inhibitor with similar size and characteristics in our analysis (Supplementary File and Fig. 1). Some inhibitor proteins can contain multiple domains and are reduced in size following proteolytic cleavage, so it’s possible Frisco strain lice may still produce the 7 kDa thrombin-inhibitor despite its presence not being obvious in our analysis.

The last inhibitor present in both salivary glands was XP_002432611.1, encoding for a putative pacifastin. The first pacifastin inhibitor was identified in the crustacean *Pacifastus leniusculus*^59^ and is a heterodimeric protein containing both heavy and light domains. The light domain contains nine consecutive inhibitory domains each containing a conserved cysteine structure consisting of: Cys_1_-Xaa_9–12_-Cys_2_-Asn-Xaa-Cys_3_-Xaa-Cys_4_-Xaa_2–3_-Gly-Xaa_3–6_-Cys_5_-Thr-Xaa_3_-Cys_6_. The P1 residue, which defines the inhibitor’s specificity, is the second residue immediately following the 5^th^ cysteine. Pacifastin-like inhibitors have been reported in multiple arthropods (crustacean, locust, mosquitoes, kissing bugs and wasps) and appear to be unique to this phylum^60^. These inhibitors may affect host immune responses^61^ including the prophenoloxidase cascade^62^. Pacifastin-like inhibitors usually contain multiple pacifastin domains, however, XP_002432611.1 only has a single pacifastin domain with a Leu residue at the P1 position, suggesting preferential inhibition of chymotrypsin-like enzymes. We speculate that this protein may contribute to blood-acquisition by interfering with the host immune response at the bite site^63^.

### Putative hormone binding proteins

We identified seven CDS encoding putative hormone-binding proteins, which were further subclassified based on their unique domains. Two of these proteins, seqSigp-61744 and seqSigp-91221, are small (13.1 kDa and 9.8 kDa, respectively) and feature an insect pheromone-binding A10/OS-D domain (PFAM 03,392). The other five CDS encode larger proteins (23–29 kDa) containing a juvenile hormone-binding domain (PFAM 06,585) within the Tubular Lipid Binding Protein (TULIP) superfamily^64^. Six of the hormone-binding protein transcripts were detected in both Bean- and U-shaped salivary glands (Supplementary file 1), while unique peptides from only four of the proteins were identified by LC–MS/MS analysis (Supplementary file 2). Notably, seqSigp-61744 and seqSigp-95885 were present in both glands, while seqSigp-91221 and XP_002432614.1 were exclusive to the protein homogenates of Bean-shaped salivary glands. Moreover, seqSigp-61744 and seqSigp-95885 were among the most abundant secreted proteins found in our LC–MS/MS data, however the transcript levels were noticeably lower, outside the top 2000 most abundant by TPM count. The observed disparity in quantification between transcripts and proteins may be attributable to a rapid transcript turnover rate.

Proteins that contain both OS-D and juvenile hormone-binding domains have been identified and characterized in non-blood feeding insects^65–67^, where they play distinct roles in insect physiology and behavior. Despite their presence in the sialome and salivary gland homogenates of mosquitoes^68,69^, these proteins are typically not overly abundant in mosquito saliva. Their potential function in blood acquisition and pathogen transmission remains undetermined.

### Putative antigen 5-like proteins

Antigen 5-like proteins are part of the cysteine-rich secretory, antigen 5, and pathogen-related 1 protein superfamily, also known as CAP proteins^70^. Antigen 5-like proteins are recognized as major allergens in wasps and hornets and may be involved in hypersensitivity responses in mammals^71,72^. We identified CDS encoding putative antigen 5-like proteins in both Bean- and U-shaped salivary glands (Supplementary file 1). However, no unique CAP peptides were detected in our LC–MS/MS analysis (Supplementary file 2), indicating low abundance of these proteins in *P. humanus* salivary glands. Despite being commonly reported in the sialome of arthropods^30,31^, their role in vector biology is not well understood. In mosquitoes, salivary antigen 5-like proteins have been reported in both males and females^73,74^, and even in mosquito species that don’t feed on blood^75^, suggesting that members of this protein family are not exclusively associated with blood-acquisition. However, in other blood-feeding vectors such as the horse fly^76^ and kissing bug^77^, antigen 5-like proteins can block platelet aggregation.

### Unknown

**Fig. 4:**
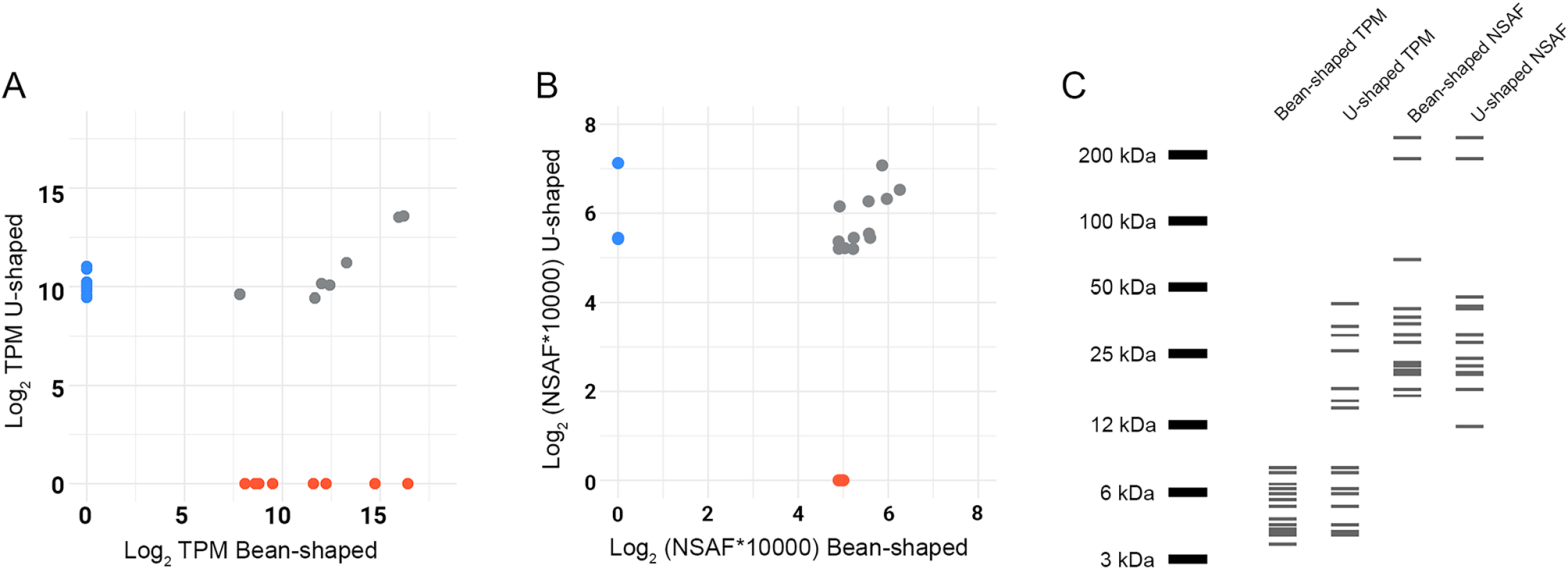
Overview of the 15 most abundant putative proteins classified as “unknown secreted” in *P. humanus* salivary glands. Scatter plot of the most abundant proteins in both Bean- and U-shaped salivary glands based on their (**A**) TPM or (**B**) NSAF values. Blue dots represent proteins found exclusively in the U-shaped gland, while orange dots denote those unique to the Bean-shaped glands. Gray dots account for proteins identified in both glands. (**C**) In silico gel presenting the 15 most abundant proteins based on their average Transcript Per Million (TPM) or Normalized Spectral Abundance Factor (NSAF).

Within the “secreted” functional class, most of the putative transcripts were classified as “unknown” for both Bean- and U-shaped salivary glands, comprising roughly 90% (Bean-shaped) and 73% (U-shaped) of the total TPM of secreted proteins (Table 1). Of the 15 most abundant “unknown secreted” sequences, 7 of the transcripts were common to both glands and shared comparable TPM levels (Fig. 4A and C). The degree of protein overlap was greater when comparing the most abundant proteins identified by LC–MS/MS, where 10 of the top 15 had similar NSAF values in both glands (Fig. 4B and C). Silver staining of salivary gland extracts also showed similar profiles of the abundant proteins between the two gland types, consistent with RNA sequencing and mass spectrometry results (Fig. 2 and supplementary Fig. 1).

One of the most abundant proteins detected in both salivary glands, but more plentiful in U-shaped glands (TPM_Bean_ = 121 and TPM_U-shaped_ = 1893), is transcript seqSigP-12505, that has nearly identical sequence to genomic entry XP_002432909.1. Unique peptides belonging to this protein were the most abundant in the U-shaped salivary glands and third most abundant for the Bean-shaped glands in the LC–MS/MS output. SeqSigP-12505 features a putative signal peptide, a projected mature protein size of 25.9 kDa, an isoelectric point of 7.78, and no glycosylation sites. AlphaFold structure prediction indicates that the mature seqSigP-12505 is organized in a bundle of alpha-helixes stabilized by two disulfide bridges (Supplementary Fig. 3). BLAST and PSI-BLAST searches across various protein and domain databases failed to retrieve any significant match to this highly abundant salivary protein (Supplementary file 3). Based on the limited information, we are unable to speculate this protein’s function, however, given its abundance in both salivary glands and its novelty to body lice, it may play an important role in blood-feeding and is a priority target for further characterization.

## Conclusion and future directions

Here we explored the protein composition of human body louse Bean- and U-shaped salivary glands. We identified several protein families commonly reported in the sialomes of other blood-feeding vectors with function related to blood acquisition. However, the majority of the CDS identified were similar to sequences of unknown function or unique to *P. humanus*, underscoring the lack of knowledge on body louse salivary proteins and the need to further study the encoded body louse salivary products.

Both salivary glands had an overall highly similar transcriptomic and proteomic profile, indicating a redundant and conserved protein composition (Figs. 2, 3, 4, and supplementary Fig. 1). This result was unexpected given the distinct morphological characteristics of the two gland types (Fig. 1) and the seemingly counterintuitive evolutionary investment of maintaining multiple pairs of salivary glands with a high degree of protein repertoire overlap. Given the rapid feeding behavior of body lice, it’s possible the glands have varying rates of secretion kinetics; where the U-shaped glands can quickly eject an essential, initial burst of saliva while the larger Bean-shaped gland produces and excretes a larger volume of saliva, albeit at a modestly slower, steadier rate. Alternatively, the two gland types may once have had more distinct salivary repertoires in human louse progenitors that parasitized chimpanzees or other primates^78^, however, in an example of convergent evolution, the glands developed increasingly redundant functions over time. Regardless, each gland type appears to contribute a limited set of unique salivary components during feeding, as we identified a modest number of putative secreted CDS (Bean = 133; U-shaped = 493) and proteins (Bean = 58, U = 14) exclusive to each gland.

Due to the lack of knowledge on the gene products of the Pawlowsky glands, and their potential involvement in transmission of *Yersinia pestis*^11^, further analysis of these unique glands is needed. Because of their small size and poor accessibility, traditional dissection techniques may not be practical for PG isolation. Exploratory, spatial proteomic or transcriptomic analysis may be useful to circumvent these challenges to begin characterizing the PG gene products.

While there is no evidence that the acini associated with the bean-shaped gland contribute to saliva production (Fig. 1), we can’t rule out this possibility given the structure’s proximal association with the other secretory glands. If so, it may expand the scope of salivary proteins produced by body lice beyond those described herein. Further characterization of the louse acini is required to determine their function in louse physiology.

## Supplementary Information

Supplementary Information 1.

Supplementary Information 2.

Supplementary Information 3.

## Data Availability

The transcriptome data was deposited at the National Center for Biotechnology Information (NCBI) under Bioproject PRJNA975842 and Biosample accession SAMN35327581, SAMN35327582, SAMN35327583 and SAMN35345087. The raw reads were deposited at the Short Reads Archive of the NCBI under accessions SRR24727591-SRR24727594. This Transcriptome Shotgun Assembly project has been deposited at DDBJ/EMBL/GenBank under the accession GLAH00000000. The version described in this paper is the first version, GLAH01000000. The raw proteomic data was deposited in the ProteomeXchange platform under accession number PXD058011. All relevant data is available within the manuscript, supplementary files, and through the accession numbers.
