# Supplementary material for: An insight into the salivary gland content of the human body louse, Pediculus humanus
Source: Sci Rep. 2025 May 26;15:18322. doi: 10.1038/s41598-025-01412-5 (PMC12106848; doi:10.1038/s41598-025-01412-5)
Supplement: Supplementary file 1 — Supplementary Information 1. [file 41598_2025_1412_MOESM1_ESM.pdf]

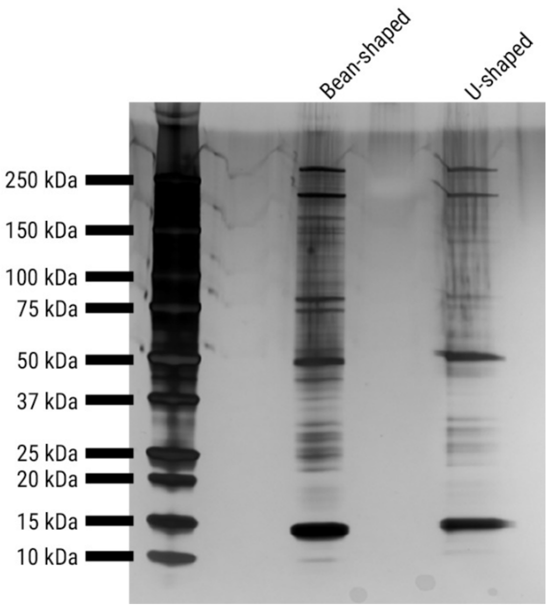

Supplementary Figure 1: Silver-stained SDS-PAGE (10%) gel containing protein extracts from Bean-shaped (5 µg) and U-shaped (3 µg) salivary glands. The Protein Precision Plus Standard (Bio-Rad) was used as the molecular weight marker.

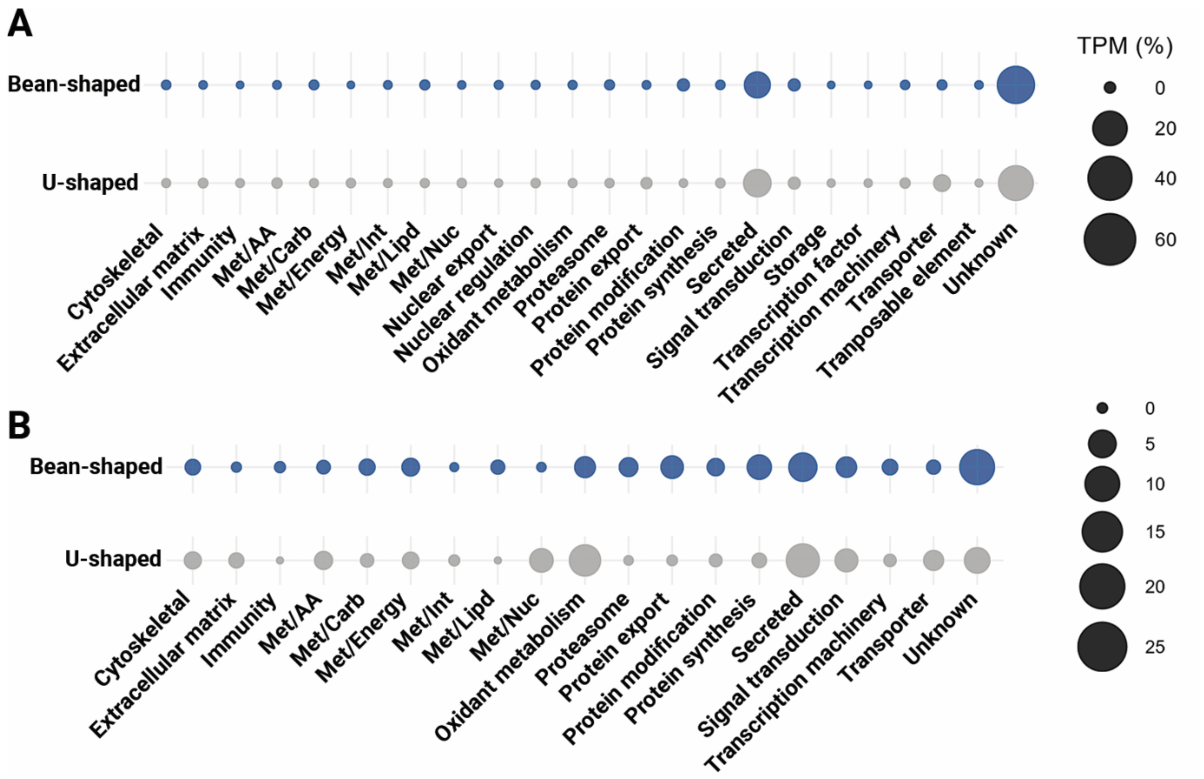

Supplementary Figure 2: Bubble plot representing the functional classification of the unique transcripts and proteins identified in the Bean- and U-shaped salivary glands of *P. humanus*. The size of the spheres represents the average **(A)** TPM of the CDS of each class or the **(B)** NSAF of the proteins identified by LC-MS/MS as percentages.

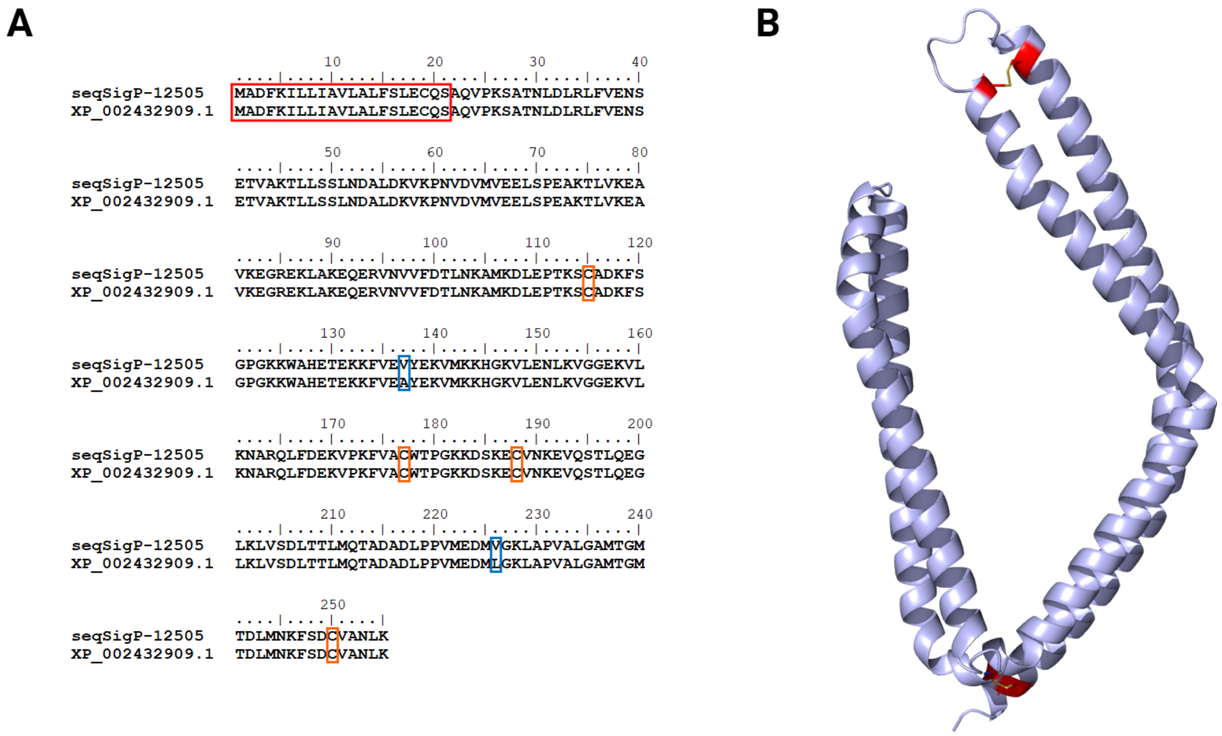

Supplementary Figure 3: Amino acid alignment of seqSigP-12505 and the *P. humanus* genomic entry XP_002432909.1. **(A)** The putative signal peptide is highlighted in red, while the cysteine residues forming the two disulfide bridges are marked in orange. Non-conserved residues between both sequences are depicted in blue. **(B)** Predicted structure of the mature seqSigP-12505 protein generated using AlphaFold2. Cysteine residues involved in disulfide bridge formation are highlighted in red. The figure was rendered and edited using PyMOL.

Supplementary File 1: Excel spreadsheet containing summary results and associated files of the transcriptome analysis.

Supplementary File 2: Excel spreadsheet containing summary results from the LC-MS analysis.
